# Moving up the On-Site Sanitation ladder in urban India through better systems and standards

**DOI:** 10.1016/j.jenvman.2020.111656

**Published:** 2021-02-15

**Authors:** Shubhagato Dasgupta, Neha Agarwal, Anindita Mukherjee

**Affiliations:** Scaling City Institutions for India, Centre for Policy Research, New Delhi, India

**Keywords:** On-site Sanitation, Regulation, Standards, Septic tanks, Public health, Water pollution, Prefabrication, India

## Abstract

Wastewater management predominantly takes the form of On-Site Sanitation (OSS) in low- and lower-middle-income countries (LMICs). In India, households construct and operate OSS systems in the absence of regulatory oversight and seldom in compliance with the national technical standards – posing a risk to water sources and public health. The present paper reviews novel evidence on the quality of these systems from a multi-state survey of 3000 households in India to identify policy and practice interventions for creating sustainable urban sanitation futures. The paper argues for local and national governments to unlock the potential of OSS as a safe and long-term wastewater management solution through (1) re-envisioning the system design to simultaneously meet household and environmental needs, (2) fostering prefabrication of systems as a means to distribute the compliance responsibility optimally, and (3) updating technical standards for facilitating such a paradigm shift.

## Highlights

• Septic tanks in urban India are non-compliant and fail to provide safe sanitation.
• High urban densities preclude subsoil dispersion as scalable solution.
• Wider adoption of prefabricated ‘packaged’ on-site systems can improve sanitation.
• Standards for on-site system performance are key to facilitate the transition.
• Greywater management needs mainstreaming within new systems, policy, and standards.

## 1 Introduction - On-Site Sanitation as the fulcrum of urban wastewater management in LMICs

**Fig. 1:**
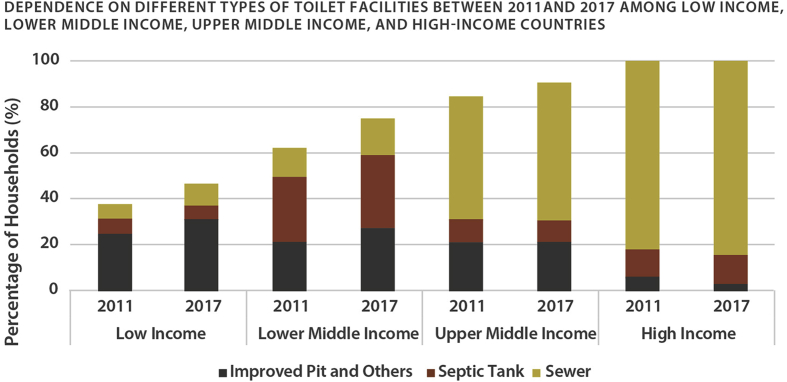
Dependence on different types of toilet facilities between 2011 and 2017 among low income, lower middle income, upper middle income, and high-income countries.

‘Messy’ and ‘hidden’ urbanisation, manifesting as rapid socio-spatial transformations that outpace the provision of basic services to the urban population, constrains the prosperity and liveability of cities across South Asia (Ellis and Roberts, 2016). Much less create centralised infrastructure for water and wastewater management, lower-middle-income countries (LMICs) like India have had to confront a high incidence of open defecation as recently as the last decade (Fig. 1). Moreover, increased access to toilet facilities has only modestly produced the benefits typically associated with sanitation and exacerbated epidemiological risks in some cases (Bancalari and Martinez, 2018; Humphrey et al., 2019; Luby et al., 2018; Odagiri et al., 2016). Citing an otherwise clear link between improved sanitation and the alleviation of adverse health outcomes, researchers impute the phenomenon to the failure of On-Site Sanitation (OSS) systems, like septic systems and leaching pits, through poor design, sighting, or maintenance (Bancalari and Martinez, 2018).

On-Site Sanitation is the mainstay of wastewater management in LMICs. In India, even as rural and urban households continue to rely on OSS systems, governmental actors widely consider these a stopgap arrangement in favour of capital- and capacity-intensive centralised sewerage systems (Prasad and Ray, 2019). However, the dependence on OSS staying near constant at 60% among urban households between 2012 and 2018 - despite a national urban infrastructure scheme underwriting sewerage development during the same period - undermines their perception as a temporary fixture of urban wastewater management (Ministry of Statistics & Programme Implementation (MOSPI), 2019). Globally, the discourse on the long-term viability of OSS as a standalone or a hybrid wastewater management solution for different spatial scales has been slowly gaining traction (Schrecongost et al., 2020).

Countries like Ireland, Malaysia, and Japan already successfully deploy OSS as part of full-scale wastewater management systems. However, any attempt at streamlining city-wide non-networked sanitation is incomplete and only partially effective without ensuring that the household-level OSS systems function well over time. The limitations of national-level datasets such as the Census of India 2011 and the National Sample Survey 2018 preclude a detailed investigation of the issues and challenges confronting OSS in an urbanizing and densifying India. With the issuing of the National Policy on Faecal Sludge and Septage Management (FSSM) in 2017, cities that have been early adopters of FSSM have typically collected detailed city-wide data on OSS systems in the form of baseline surveys. Nonetheless, these baseline surveys remain siloed within their city-level context and have not found broader application.

In this context, the authors of the present paper undertook a novel sample survey on OSS systems – interviewing 3000 households and over 50 non-household actors (including masons, public desludging operations, city government engineers, among others) in ten cities across four hydrogeologically-diverse states in India (Dasgupta et al., 2019). The present paper is the first attempt to review novel multi-state evidence on the quality of OSS systems for identifying national-level policy interventions to create sustainable sanitation futures for urban India. In creating the roadmap, the authors have also considered the wider regulatory landscape, markets, and standards that comprise the OSS ecosystem in the country. The recommendations laid out in the following sections while rooted in the Indian context may also have applicability to other LMICs given the similarity in challenges confronting the effective implementation and management of OSS (Hashimoto, 2019).

## 2 Deliberate innovation in system design necessary to respond to contemporary ground realities

The septic tank is a primary treatment unit that effects a reduction of 30–50% in the biochemical oxygen demand (BOD), and acts minimally on pathogens and the nutrient load. Consequently, the effluent from the tank needs further remediation through subsoil dispersion systems or impermeable secondary and tertiary treatment units (Tilley et al., 2014). In a conventional septic tank system, also called ‘septic system’, a subsoil dispersion system such as the soak pit or dispersion trenches follow the septic tank. On the other hand, leaching pits, primarily ideal for rural or low-density settings, directly discharge wastewater from the dwelling unit into the surrounding subsurface for remediation.

**Fig. 2:**
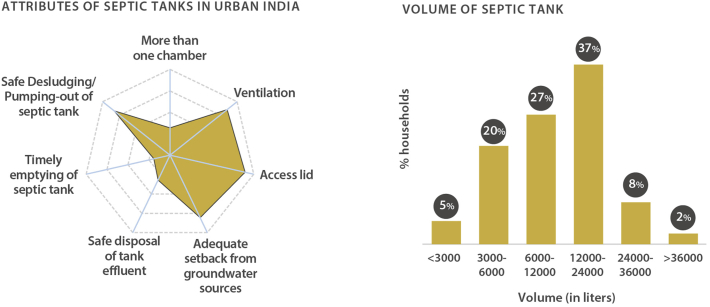
Septic tanks exhibit deviation from the governing standard in all key aspects resulting in their significant non-compliance and variation on-ground. About half the septic tanks are more than 12,000 L in volume.

Septic systems are a widely-accepted safe alternative to networked sanitation in peri-urban and rural areas subject to factors like hydrogeology, maintenance of sufficient setback distance from any groundwater sources in the vicinity, and the cumulative pollutant loading in the local environment. But as per survey findings, 72% of septic tanks in urban India discharge inadequately treated wastewater directly into stormwater drains (Dasgupta et al., 2019) (Fig. 2). Previous studies find that such a lack of effluent management not only leads to pollution of water bodies but is also associated with an increased risk of diarrhoea among children under five (Bancalari and Martinez, 2018).

The substitution of a septic tank for a septic system is common in parlance and practice. Just as the design of the conventional septic tank system, the deviation has emerged as a function of ‘construction convenience, low cost, and repetitive practice’ (Dasgupta et al., 2019; Victor A. D'Amato, 2008). Current thinking posits one of two solutions to these challenges – retrofitting septic tanks with soak pits, or implementing city-wide off-site effluent management. The following sections show that both these options while potentially feasible in specific contexts are not scalable in urban settings.

### 2.1 The soak pit challenge

The soak pit proposition encounters difficulties at two levels in urban areas. In coping with the unavailability of reliable desludging services and in a bid to avoid not only the related costs but also the physical engagement with the septic tank, households prefer larger septic tanks that require less frequent desludging. In a clear trade-off between capital costs and O&M costs, both households and masons consider a septic tank that doesn't require emptying for a 40-50 year-long period the gold standard. Resultingly, the average capacity of a septic tank among urban households is 13,375 L (Dasgupta et al., 2019).

Larger tanks typically benefit from compartmentation to avoid resuspension of solids and better settling which helps minimise particle entrainment and clogging of soak pits (D'Amato, 2008). However, as many as 67% of septic tanks larger than 2,000 L (the threshold for compartmentation requirement in the Indian technical standard) are single-chambered and thus inefficient for their size (Dasgupta et al., 2019). Therefore, while managing effluent through soak pits in these cases would require not only the construction of a subsoil dispersion system, but also the in-situ rehabilitation of the primary unit. However, about 80% of households report that their septic tanks are located directly underneath the toilet or building, and nearly 50% of the households claim that their dwelling unit is fully built-up (Dasgupta et al., 2019). Retrofitting the septic tank with a soak pit under these circumstances would lead to significant disruption for the household as a direct challenge to its at scale and in a timely manner.

Second, and more importantly, despite favourable preconditions, their large-scale implementation is infeasible due to the very nature and working principle of soak pits. Even when space is present for the construction of a subsoil dispersion system, it cannot accompany all septic tanks at the urban neighbourhood or city scale. A significant body of research across time and geographies has found a positive correlation between the density of leaching-based OSS systems and groundwater contamination in a region (Arwenyo et al., 2017, Bicki and Brown, 1991, Yates, 1985). Research studies focused on the role of density in India, although limited, show that groundwater sources in densely toileted areas are significantly more contaminated than those with open defecation (Bhallamudi et al., 2019). High system density, as encountered in urban settings, compromises both the soil carrying capacity and the ability to maintain appropriate setback from groundwater sources.

**Fig. 3:**
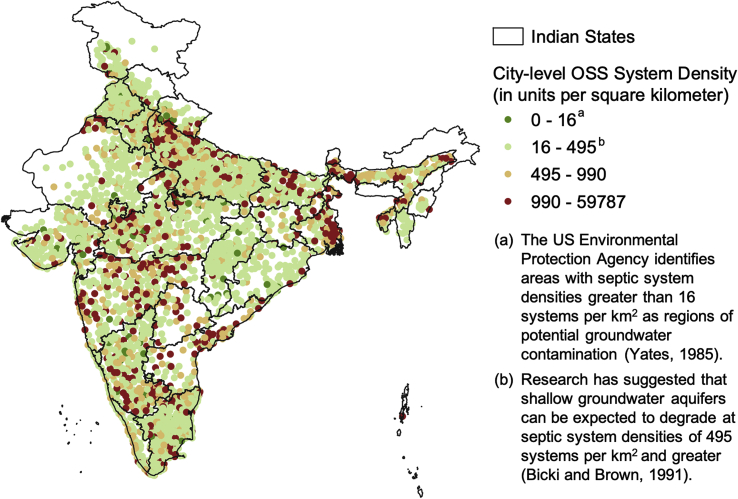
City-level density of OSS systems (septic tanks and leaching pits) in urban India (each point represents an individual city/town).

The threshold varies widely depending on the context, but ranges from as low as 16 septic systems per square kilometre to 495 subsoil dispersion systems per square kilometre (Bicki and Brown, 1991, Yates, 1985). These density thresholds are exceeded at the city scale in urban India and run the risk of being even higher at the neighbourhood scale (Fig. 3). The issue is especially pernicious for smaller cities and towns that concomitantly rely on OSS and a groundwater-based water supply regime (Dasgupta et al., 2019; Kulkarni and Shah, 2015).

### 2.2 The off-site effluent management challenge

Consecutive five-year programmes for large-scale urban infrastructure development, like the latest Atal Mission for Rejuvenation and Urban Transformation (AMRUT), have focused only on the biggest cities in India. Sewerage and septage management made up 42% of AMRUT's total outlay of INR 77,640 crores (USD 10.25 billion) (Dasgupta et al., 2020). Still, by the end of the programme, urban local bodies had implemented projects worth only 13% of the outlay. Dedicated city-wide off-site effluent management is an undertaking of a similar scale and made more challenging in smaller cities and towns due to lower technical and financial capacities of urban local bodies (Ministry of Housing and Urban Affairs, 2020; Nath, 2019). In the short-to medium-term, covering the drains along with the interception and in-situ treatment of wastewater might alleviate some of the public health and environmental concerns. However, the dual use of drainage infrastructure - not designed for this purpose - may compromise its primary function of stormwater management.

Like ‘sewered’ and ‘non-sewered’, binaries between ‘all soak pits’ and ‘no soak pits’, or ‘small-bore sewerage everywhere’ and ‘small-bore sewerage nowhere’ should not bind a city-level sanitation strategy for effluent management. Accordingly, examining the challenges associated with implementing these effluent management solutions at city-scale does not rule out their feasibility and appropriateness at the sub-city or neighbourhood scale. Nonetheless, it must be recognised that the conventional system design based on subsoil dispersion is inherently incompatible with dense urban settings. Even if effective and feasible in specific settings, subsoil dispersion, off-site effluent management, or in-situ retrofitting of a septic tank, are not scalable and efficient solutions within a reasonable timeframe.

Countries like Malaysia and Japan have phased out subsoil dispersion systems as the go-to solution in urban contexts in favour of packaged systems that achieve at least secondary treatment at the household-level in Malaysia and tertiary treatment in Japan. The resulting effluent is dischargeable to the environment without compromising public health safety and reusable, depending on the nature of application and the extent of treatment. Therefore, higher levels of in-situ wastewater treatment can help secure sanitation outcomes even when soak pits and dispersion trenches are not feasible to implement. Despite emerging research on novel systems and the availability of such options in India, neither their need nor application are yet mainstream (Sharma and Kazmi, 2016; Vanwonterghem et al., 2018). Therefore, going forward, the government needs to recognise and respond to contemporary ground realities by strongly pursuing and deploying innovation in system design.

## 3 Industry-scale prefabrication of OSS systems can overcome limitations in local regulatory and technical capacities

As seen earlier, the OSS trajectory in LMICs must advance to packaged systems that achieve high enough in-situ treatment of wastewater to not require city-wide capture and off-site management of effluent. However, introducing such a shift under the current paradigm of in-situ construction with the oversight of urban local bodies meets with distinct and foreseeable challenges. These include (1) large-scale retraining of masons and (2) capacity building of urban local bodies to ensure that masons and households act desirably to uphold the public (and private) good.

The bundle of ‘wicked problems’ - preceding the actual intervention – require sustained financial and social investments. Moreover, given the trap of premature load-bearing of institutional systems, the investments may not offer guaranteed returns over a predictable timeframe (Pritchett and Weijer, 2010). Urban local bodies in India grapple with a shortage of staff, inadequate skills and capacity of existing personnel, and a lack of state-level resource and support institutions (National Institute of Urban Affairs, 2015). However, the weakness of the building plan approval process, which comprises an evaluation of OSS system, has been attributed to not only weak capacity, but also to the poor accountability of institutions and rent-seeking opportunities (Singh, 2018). With the existing nexus between the urban local body, masons, and households not bearing the desired results of compliance and quality of OSS systems, it is important to re-evaluate and reorganise stakeholder roles.

**Fig. 4:**
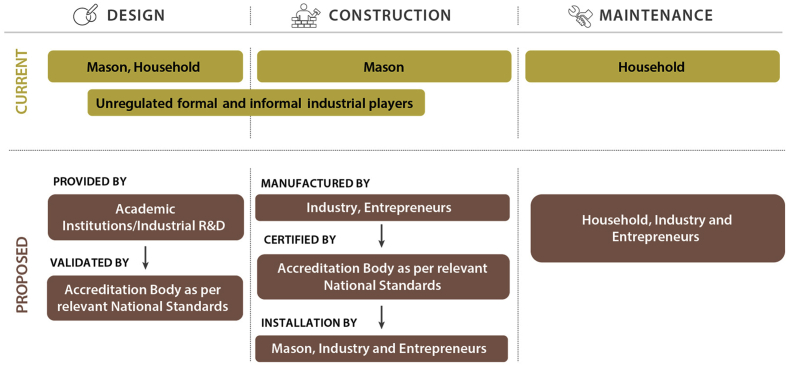
A proposed framework for reorganising OSS system-related roles and responsibilities among the various stakeholders.

Prefabrication of OSS systems offers the opportunity to assign the responsibilities for ensuring standardisation of system design at the national or state level instead. Many industrial players, ranging from regional to national, already ply the market with prefabricated ‘septic tanks’ but are currently unregulated (barring precast concrete septic tanks). The issue also persists among small-scale sewage treatment plants (SSTP), prescribed by Ministry of Environment, Forests, and Climate Change (MoEFCC), for buildings with a total built-up area above 20,000 square metres (Reymond et al., 2020). Therefore, the regulators at the national and state level should strive to formalise and regulate the prefabricated OSS system and SSTP industries, and facilitate a transition from conventional OSS configurations to more advanced packaged systems (Fig. 4). Industries can leverage an assembly-line approach to create certifiably standardised systems and, with the right set of incentives, innovate in design. Unsurprisingly, advancements in system designs have been accompanied by industrial prefabrication in countries with a more mature, safer, and sustainable OSS ecosystem (Dasgupta et al., 2019; Hashimoto, 2019).

Prefabricated packaged systems can supplant in-situ construction of the OSS system with minimal disruption to the overall construction process while bringing benefits of standardised design, faster installation, greater performance, and lesser environmental and public health costs generally associated with prefabrication of building infrastructure (Cheng et al., 2014; Jaillon and Poon, 2008). Since environmental costs are often not accounted for within systems and processes and given that the OSS system prefabrication industry in India is still small, prefabrication of OSS system may not be cost-competitive with in-situ construction from a household's perspective. Masons interviewed as part of the survey reported that a septic tank costs INR 3.2 per litre on average, or in other words, that a five cubic meter septic tank would cost INR 16,000 (USD 217). In comparison, a similarly-sized rotomolded polyethylene septic tanks, averaging INR 16.3 per litre, can cost an estimated INR 81,500 (USD 1,106).

Therefore, governmental support in the form of (1) mandating packaged systems for government and institutional buildings, and (2) offering financial assistance to households for OSS system upgradation (like Japan and Ireland), especially in environmentally sensitive regions, can expedite scale-up and lower costs for both manufacturers and households.

## 4 Regulatory interventions are imperative to moving the sector forward

Non-compliance with the governing technical standards and the nature of resulting deviations in OSS systems evinces the misalignment between the expectations of households and regulators. The regulator is expected to desire the safeguarding of environmental and public health through consistently well-performing systems. On the other hand, the household prioritises minimising future costs and hassles associated with the management of the OSS system. Households achieve this, within the bounds of their resources, through constructing excessively large and single-chambered septic tanks and disposing effluent into drains instead of a secondary treatment or subsoil dispersion system. The existing Indian technical standards for septic tank systems have not been substantively updated since their issue in the 1980s and accordingly, fail to realign regulation with contemporary realities and concerns. For instance, although local and regional entrepreneurs and industries have been supplying prefabricated septic tanks to the market, they are currently unregulated owing to a lack of a nationally-issued technical standard.

A revamping of national technical standards underpins the paradigm shift discussed in section 3 - paving the way for sectoral reforms and providing a clear trajectory for improvements to system design.

### 4.1 Resolving the density conundrum

Unlike developed countries, LMICs depend on OSS for both urban and rural wastewater management. Differences in settlement density and regional hydrogeological specificities combine to create a high diversity of contexts for implementing OSS. Noting the correlation between system density with adverse public health outcomes and the contamination of water sources, researchers have deemed it the overriding criteria for preventing pollution and argued for regulators to define a standard for density (Borchardt et al., 2003; Yates, 1985). Whereas subsoil dispersion systems may still be effective as a low-cost effluent disposal mechanism in rural and peri-urban areas, urban areas require a comprehensive site assessment to determine the suitability of such a system and safe alternatives to subsoil dispersion, where not unsuitable.

Regulators have adopted two approaches to accounting for system density. The first approach specifies the minimum plot size for soak pit feasibility, or conversely, the number of units per unit area with estimates stating that the minimum plot size to prevent contamination is half to one acre or 21,780 to 43,560 square feet (Perkins, 1984). The second, as espoused by the Irish Environmental Protection Agency, undertakes a case-by-case evaluation to determine the impact of an additional subsoil dispersion system in a given area on the cumulative nitrogen loading (Ireland Environmental Protection Agency, 2010).

The second approach requires significant local-level technical expertise and capacity, but the first allows the estimation of the suitable density thresholds for a region by modelling hydrogeological considerations. The applicable density thresholds can then be situated along with key factors like the type and size of a settlement, regional hydrogeological vulnerability, and others in a national or state level framework for sanitation planning. State-level regulators like the Pollution Control Bodies, Public Health Engineering Departments, Water Supply and Sewerage Boards can use such a framework to guide local bodies in selecting the appropriate sanitation system for the protection of public and environmental health – whether an advanced ‘packaged’ treatment system, a septic system, twin leaching pits, or a centralized sewerage system.

### 4.2 Benchmarking performance

Where subsoil dispersion systems are not feasible, several countries specify the quality of effluent that must be met to allow its discharge into the open environment. The Indian standard categorically states that ‘under no circumstances shall effluent from a septic tank be allowed into an open channel drain without adequate treatment’. Accordingly, it also discusses non-subsoil dispersion systems for managing effluent, but it does not specify any quality criteria which the effluent must meet for considering it safe for disposal (Bureau of Indian Standards, 1985).

Existing Indian standards for wastewater treatment are specifically applicable only to ‘sewage treatment plants’ and subject to frequent revision. A regulatory lacuna still prevails along the non-networked sanitation service chain, including for the reuse of biosolids (Agarwal et al., 2020; Schellenberg et al., 2020). The absence of effluent renders prefabrication a suboptimal exercise without a clear performance benchmark for researchers and industrial players to strive towards. Additionally, issuing performance specifications also mainstreams the inherent objectives of OSS systems as components of wastewater management rather than a mere containment unit for faecal waste. It is only through the institution of a specific metric that the performance of an OSS system can be measured and reported in a credible, transparent, and consistent manner.

International frameworks like the ISO's ‘Non-sewered sanitation systems — Prefabricated integrated treatment units — General safety and performance requirements for design and testing’ often attempt to leapfrog from a basic service level to highly advanced sanitation systems (International Organization for Standardization, 2018) (Table 1). This goal tends to be elusive in LMICs where incremental approaches more strongly favour universal coverage and sustainability of sanitation services. Since households across the socioeconomic spectrum are responsible for the capital and operating costs of an OSS system over its lifetime, any performance standard for these systems must balance costs of compliance with the benefits to public health and safety. Although wastewater, or more specifically blackwater, management *de facto* has been stuck at primary treatment at the household-level, national regulators like the Central Pollution Control Board can devise a graduated needs-based standard for OSS system performance. The standard, specifying different level of OSS system performance, can directly feed into the national planning framework discussed earlier.

**Table 1:** Comparison of wastewater treatment standards and septic tank effluent characteristics.

Parameter	Maximum Concentration in Liquids								
	ISO 30500	India							
		General Discharge Standards				MoEFCC Notification, October 2017		NGT order 2019 (for mega and metropolitan cities)	Average Septic Tank Effluent Characteristics (based on a sample of 32 septic tanks**)
		Inland surface water	Public sewers	Land irrigation	Marine coastal areas	Metro cities and specific state capitals	All other regions		
Biochemical Oxygen Demand (mg/l)						20	30	10	203
Chemical Oxygen Demand (mg/l)	≤ 50 for Category A and ≤ 150 for Category B*	250			250			50	619
Total Suspended Solids (mg/l)	≤ 10 for Category A and ≤ 30 for Category B*	100	200	600		50	100	20	2,377
Total Nitrogen (% load reduction)	70								
Total Kjeldahl Nitrogen (mg/l)		50	100		100			10	318
Dissolved Phosphorous (mg/l)		5						1	
Total Phosphorous (% load reduction)	80								
pH	6–9					6.5–9	6.5–9		6.69
*E. coli* (CFU/l)	100 (≥6 log reduction)	N.A.							
MS2 Coliphage (PFU/l)	10 (≥7 log reduction)								
*Ascaris suum* viable ova (#/l)	<1 (> = 4 log reduction)								
viable Clostridium perfringens spores (CFU/l)	<1 (> = 6 log reduction)								
Faecal Coliform (MPN per 100 ml)						1,000	1,000	230	1.63 × 10^7^ CFU per 100 ml

*Category A comprises unrestricted urban uses where public access is not restricted (e.g. landscape irrigation, toilet flushing) and Category B usage refers to discharge into surface water and other restricted urban uses that comprise all uses where public access is controlled or restricted by physical or institutional barriers (e.g. fences, temporal access restriction).

**Authors' study

### 4.3 Rethinking greywater management

The production of greywater, or non-toilet related wastewater from bathing, washing, and other such households activities, varies in response to the nature of water supply, type of water and sanitation-related infrastructure, culture and habits, among others, but can comprise up to 90% of a household's wastewater load (Oteng-Peprah et al., 2018; Shaban and Sharma, 2007). Containing residue from pharmaceuticals, personal care products, aerosols, and pigments, greywater is a source of localised pollution, especially micropollutants (Moilleron et al., 2017). The World Health Organization also recognises the potential of greywater to transmit disease (World Health Organization, 2006).

While a centralised sewerage system conveys both blackwater and greywater to an off-site treatment facility, urban households dependent on OSS systems report discharging greywater directly into stormwater drains (Dasgupta et al., 2019). Interestingly, the national technical standard for the design and installation of septic tanks currently recommends that ‘wastes containing excessive detergents, grease and disinfectants should not be treated in septic tank as they adversely affect the anaerobic decomposition’ (Bureau of Indian Standards, 1985). Given any city-wide sanitation solution is incomplete without addressing greywater management, the standard needs revising to allow for the development of novel OSS systems that successfully co-manage blackwater and greywater.

## 5 Conclusion

The Sustainable Development Goal 6 requires nations to ensure universal access to safely managed sanitation services at the household-level and the halving of untreated wastewater by 2030. The construction of new toilet facilities, and sewage and septage treatment infrastructure is not enough to meet these targets. Ensuring the proper performance of systems and infrastructure at each step of its service chain is imperative to achieving safely managed sanitation. Despite its critical role as a localized wastewater management system, an OSS system is typically viewed as falling outside the purview of mainstream planning and governance systems. Noting the continually strong dependence of populations on OSS across LMICs, including India, it is necessary to plan long-term improvements to the household-level infrastructure for achieving desired sanitation outcomes.

Evidence suggests that the wide-ranging non-compliance in the design of OSS systems notwithstanding, their conventional configurations, even when properly designed, are unsuited to dense urban settings. Recognising the limitation, packaged systems should supplant the septic system or other subsoil dispersion-based systems as the default choice in on-site sanitation technologies in the medium- to long-term. National regulators can deploy the expertise of industries engaged in the manufacture of prefabricated septic tanks, as well as, research institutions to advance research and manufacture of packaged systems. Updated technical standards incorporating performance benchmarking are essential for facilitating these transitions. Similarly, a robust national regulatory framework can enable more informed sub-national action for the selection and installation of the appropriate sanitation system. Together, these systemic changes, within a multi-pronged strategy, have the potential to realign the ground realities of OSS systems with their treatment expectations in a scalable, efficient, and reliable manner.

## Credit author statement

Shubhagato Dasgupta: Conceptualization, Writing - review & editing, Funding acquisition, Supervision, Neha Agarwal: Formal analysis, Writing - original draft, Writing - review & editing, Visualization, Anindita Mukherjee: Conceptualization, Writing - review & editing, Project administration, Supervision.

## Declaration of competing interest

The authors declare that they have no known competing financial interests or personal relationships that could have appeared to influence the work reported in this paper.
